# Endocrine Characteristics of 246 Cases of Childhood-Onset Anorexia Nervosa in Japan: A Single-Center Experience

**DOI:** 10.7759/cureus.91166

**Published:** 2025-08-28

**Authors:** Yuji Oto, Takeshi Inoue, Ryoko Otani, Naho Matsushima, Tasuku Kitajima, Ayako Shiihashi, Akihisa Nitta, Satomi Koyama, Tomozumi Takatani, Ryoichi Sakuta

**Affiliations:** 1 Pediatrics, Dokkyo Medical University Saitama Medical Center, Saitama, JPN; 2 Child Development and Psychosomatic Medicine Center, Dokkyo Medical University Saitama Medical Center, Saitama, JPN

**Keywords:** anorexia nervosa, central hypogonadism, growth hormone resistance, non-thyroidal illness, pediatric

## Abstract

Anorexia nervosa (AN) is associated with multiple endocrine abnormalities resulting from chronic food deprivation due to patients’ persistent efforts to maintain thinness. Most available data are derived from adult populations, whereas pediatric data remain relatively limited. We aimed to evaluate the endocrinological characteristics of children with AN. We conducted a retrospective, single-center study of 246 Japanese patients with AN who met the eligibility criteria between January 2013 and December 2022. Physical characteristics were collected, and their correlations with various endocrinological measurements were analyzed. Insulin-like growth factor I standard deviation score (IGF-1 SDS) was positively correlated with body mass index SDS (BMI SDS) (R = 0.405, p < 0.0001) and negatively correlated with growth hormone (GH) (R = −0.424, p < 0.0001). Despite elevated GH secretion, IGF-1 levels were low, indicating GH resistance. Free triiodothyronine was positively correlated with BMI SDS (R = 0.381, p < 0.0001). Moreover, luteinizing hormone and follicle-stimulating hormone levels were positively correlated with BMI SDS (R = 0.365, p < 0.0001 and R = 0.409, p < 0.0001, respectively). Adrenocorticotropic hormone (ACTH) showed no correlations with BMI SDS (R = −0.031, p = 0.677), whereas cortisol was negatively correlated with BMI SDS (R = −0.321, p = 0.0107). In summary, this study of 246 patients with childhood-onset AN demonstrated that children, similar to adults, exhibit GH resistance and central hypogonadism. However, unlike in adults, ACTH elevation was not observed.

## Introduction

Anorexia nervosa (AN) is a psychiatric disorder that predominantly affects women and adolescent girls. It is characterized by a distorted body image, self-induced starvation, excessive weight loss, and an intense pathological fear of fat [1].

In Japan, the prevalence of AN among girls in elementary, junior high, and senior high schools is estimated at 0%-0.10%, 0%-0.40%, and 0.05%-0.56%, respectively [2]. By comparison, a systematic review and meta-analysis reported pooled incidence rates of AN in women ranging from four to eight per 100,000 person-years in North America and Europe, with generally lower rates observed in Asia [3]. These findings indicate that AN is not a rare disorder, and the number of young patients diagnosed before the onset of menstruation is increasing [2]. Furthermore, the coronavirus disease 2019 (COVID-19) pandemic has contributed to a rise in both the prevalence and hospital admission rates of AN among juveniles worldwide.

AN is associated with multiple endocrine abnormalities, primarily as a consequence of chronic food deprivation resulting from patients’ persistent drive to maintain thinness [4-6]. Chronic nutritional deprivation induces both physical and psychological stress, leading to activation of the hypothalamic-pituitary-adrenal (HPA) axis. Elevated corticotropin-releasing hormone and cortisol suppress gonadotropin-releasing hormone secretion, causing hypogonadotropic hypogonadism, and directly decrease appetite and food intake, thereby exerting a secondary anorectic effect [4]. In addition, reduced leptin levels together with increased ghrelin and cortisol during starvation suppress gonadotropin secretion, manifesting clinically as hypothalamic amenorrhea [5,6]. Despite elevated growth hormone (GH) levels in patients with AN, insulin-like growth factor I (IGF-1) levels are reduced compared with healthy controls [4-6].

To date, most knowledge of AN is derived from studies in adults, whereas pediatric data remain limited. Therefore, we aimed to evaluate the endocrinological characteristics of children with AN. We hypothesized that pediatric patients would exhibit endocrine changes similar to those documented in adults with AN.

## Materials and methods

This was a retrospective single-center study of 246 Japanese patients with AN. Patients diagnosed with AN and treated at Dokkyo Medical University Saitama Medical Center between January 2013 and December 2022 were included. This retrospective study evaluated the clinical and endocrinological characteristics of elementary and junior high school children. Patients with AN were diagnosed and classified based on clinical criteria consistent with the Diagnostic and Statistical Manual of Mental Disorders, Fifth Edition (DSM-5) [7]. Individuals with avoidant/restrictive food intake disorder (ARFID) were excluded from this study.

Disease severity was determined using the age-specific criteria of the Japanese Society for Pediatric Endocrinology. Severity was classified as mild (<85% to 75% of standard body weight), moderate (<75% to 65%), and severe (<65%) [8]. Patients whose body weight at presentation was ≥85% of the standard body weight were categorized as “normal weight” for descriptive purposes. This group included individuals who, despite appearing to have a normal body weight at admission, had experienced substantial recent weight loss, including those who had been overweight or obese prior to disease onset. The admission criteria included one or more of the following: moderate or severe malnutrition (<75% standard body weight), hypothermia, hypotension, electrocardiographic abnormalities, or electrolyte disturbances.

In this study, we evaluated physical and endocrinological differences depending on disease severity. We evaluated the height, weight, and body mass index (BMI) at the time of initial examination and noted amenorrhea in women. Regarding amenorrhea, the average age of menarche in Japan is reported as 12.3 years [9], and children aged <12.3 years were excluded. Menstrual status was evaluated only in female patients aged ≥12.3 years, as younger patients may not have reached menarche. Of the 246 total patients, 11 were male and 73 were female aged <12.3 years, leaving 162 patients eligible for this analysis.

Blood tests included IGF-1, GH, thyroid-stimulating hormone (TSH), free thyroxine (FT4), free triiodothyronine (FT3), luteinizing hormone (LH), follicle-stimulating hormone (FSH), estradiol (E2), adrenocorticotropic hormone (ACTH), and cortisol. The serum IGF-1, GH, TSH, FT4, FT3, LH, FSH, E2, ACTH, and cortisol levels were measured using electrochemiluminescence immunoassays (ECLIA) with a Cobas e801 analyzer (Roche Diagnostics K.K., Tokyo, Japan). To account for age and sex differences, height, weight, BMI, and IGF-1 levels were converted into standard deviation scores (SDS), following the approach reported in previous studies [10,11].

All statistical analyses were performed using EZR (Saitama Medical Center, Jichi Medical University, Saitama, Japan), a graphical user interface for R (The R Foundation for Statistical Computing, Vienna, Austria). EZR is a modified version of the R Commander, which adds statistical functions for frequently used biostatistical analyses. All central tendencies are expressed as medians (interquartile ranges). Comparisons between three or more groups were performed using the Kruskal-Wallis test. Spearman’s rank correlation coefficient was used for correlation analysis. p-values <0.05 were considered statistically significant. p-values <0.0001 were considered highly statistically significant. This study was approved by the Ethics Committee of Dokkyo Medical University Saitama Medical Center (reference numbers #1336, #1880). Written informed consent was obtained from all participants and their parents. All experiments were conducted in accordance with the tenets of the Declaration of Helsinki.

## Results

Clinical characteristics of patients at the first visit or hospital admission for AN

Between January 2013 and December 2022, 390 patients were diagnosed with an eating disorder at our institution. Of these, 131 patients met the diagnostic criteria for ARFID and were excluded. An additional 13 patients were excluded owing to the lack of informed consent or substantial missing data. The remaining 246 patients with AN who met the eligibility criteria were included. Mild, moderate, and severe cases accounted for 49 (19.9%), 104 (42.3%), and 63 (25.6%) of the cases, respectively. Patients were predominantly female (95%). The median age of admission or first visit was 13.6 (12.7-14.6) years, and no correlation with severity of illness was observed. Additionally, among the 162 female patients aged ≥12.3 years, 158 (97.5%) had amenorrhea. Male patients (n = 11) and female patients aged <12.3 years (n = 73) were excluded from this calculation (Table 1).

**Table 1:** Baseline characteristics of patients with anorexia nervosa. BMI: body mass index; SDS: standard deviation score p-values of <0.05 were considered statistically significant. p-values of <0.0001 are highly statistically significant. *p < 0.05; **p < 0.0001. ¹158 out of 162 patients ²11 out of 12 patients ³28 out of 29 patients ⁴69 out of 71 patients ⁵50 out of 50 patients

	Total	Normal	Mild	Moderate	Severe	p-value
Case, n (%)	246	30 (12.2%)	49 (19.9%)	104 (42.3%)	63 (25.6%)	
BMI SDS	−3.41 (−4.34~−2.38)	−0.86 (−1.47~−0.13)	−2.05 (−2.39~−1.67)	−3.39 (−3.81~−2.95)	−4.93 (−6.13~−4.50)	<0.0001**
Sex, female/male (% female)	235/11 (95.5%)	28/2 (93.3%)	45/4 (91.8%)	101/3 (98.1%)	61/2 (96.8%)	<0.0001**
Age of admission or first visit (years)	13.6 (12.7~14.6)	13.4 (12.4~14.0)	13.4 (12.4~14.6)	13.7 (12.9~14.8)	13.6 (12.9~14.4)	0.2020
Admission/outpatient (% admission)	141/105 (57.3%)	8/22 (26.7%)	13/36 (26.5%)	67/37 (64.4%)	53/10 (84.1%)	<0.0001**
Amenorrhea	158 (97.5%)¹	11 (91.7%)²	28 (96.6%)³	69 (97.2%)⁴	50 (100%)⁵	0.3544

Endocrine characteristics at first visit or hospital admission for AN

The median IGF-1 SDS value was −5.22 (−7.18 to −3.53). IGF-1 SDS decreased with increasing severity. Conversely, the median GH value was 4.82 (2.06-9.92: normal range, 0.13-9.88) ng/mL and increased with increasing severity. The median FT4 and FT3 values were 0.97 (0.89-1.09: normal range, 0.90-1.70) ng/dL and 1.57 (1.02-2.08: normal range: 2.30-4.00) pg/mL, respectively. The FT4 and FT3 levels decreased with increasing severity. LH, FSH, and E2 were 0.1 (0.1-0.4) mIU/mL, 0.7 (0.3-3.5) mIU/mL, and <5.0 (<5.0-9.9) pg/mL, respectively, and decreased with increasing severity. In the adrenal system, the ACTH levels were 15.5 (11.3-20.9: normal range: 7.2-63.3) pg/mL and were almost constant regardless of severity, while the cortisol levels were 14.3 (11.0-19.4: normal range: 7.07-19.60) µg/dL and increased with severity progression (Table 2).

**Table 2:** Clinical data of patients with anorexia nervosa. Data are shown as median (interquartile range). ACTH: adrenocorticotropic hormone; BMD: bone mineral density; E2: estradiol; FSH: follicle-stimulating hormone; FT3: free triiodothyronine; FT4: free thyroxine; GH: growth hormone; IGF-1: insulin-like growth factor I; LH: luteinizing hormone; SDS: standard deviation score; TSH: thyroid-stimulating hormone p-values of <0.05 were considered statistically significant. p-values of <0.0001 are highly statistically significant. *p < 0.05; **p < 0.0001

	Total	Normal	Mild	Moderate	Severe	p-value
IGF-1 SDS (n = 184)	−5.22 (−7.18~−3.53)	−5.09 (−6.86~−4.04)	−5.06 (−3.56~−2.49)	−5.15 (−7.03~−3.36)	−6.47 (−8.58~−5.10)	<0.0001**
GH (ng/mL) (n = 184)	4.82 (2.06~9.92)	3.43 (1.73~4.82)	2.76 (1.18~5.68)	5.04 (2.47~13.30)	7.20 (3.21~13.33)	0.0014*
TSH (µIU/ml) (n = 230)	1.58 (1.01~2.37)	1.65 (1.18~2.34)	1.57 (0.90~2.36)	1.47 (0.99~2.24)	1.83 (1.20~2.71)	0.2150
FT4 (ng/dL) (n = 230)	0.97 (0.89~1.09)	1.04 (0.94~1.20)	0.99 (0.92~1.11)	0.97 (0.89~1.06)	0.93 (0.82~1.08)	0.0293*
FT3 (pg/mL) (n = 230)	1.57 (1.02~2.08)	1.93 (1.56~2.65)	1.95 (1.57~2.70)	1.45 (0.95~2.00)	1.24 (0.86~1.63)	<0.0001**
LH (mIU/mL) (n = 225)	0.1 (0.1~0.4)	0.4 (0.1~6.4)	0.3 (0.1~1.3)	0.1 (0.1~0.3)	0.1	<0.0001**
FSH (mIU/mL) (n = 225)	0.7 (0.3~3.5)	3.1 (0.8~6.0)	2.6 (0.6~5.7)	0.7 (0.3~2.9)	0.4 (0.2~1.0)	<0.0001**
E2 (pg/mL) (n = 223)	<5.0 (<5.0~9.9)	6.8 (<5.0~25.7)	<5.0 (<5.0~17.4)	<5.0 (<5.0~9.5)	<5.0 (<5.0~6.6)	0.0113*
ACTH (pg/mL) (n = 186)	15.5 (11.3~20.9)	11.1 (6.9~23.0)	14.6 (12.1~17.9)	16.0 (12.0~20.8)	16.5 (11.3~23.2)	0.6280
Cortisol (µg/dL) (n = 186)	14.3 (11.0~19.4)	10.6 (9.5~17.7)	10.3 (8.3~14.3)	14.3 (11.9~18.0)	16.9 (13.9~21.3)	<0.0001**
BMD SDS (n = 131)	−0.80 (−1.95~0)	−1.75 (−2.13~−0.10)	−2.18 (−1.55~0.25)	−0.55 (−1.55~0)	−1.10 (−1.90~−0.20)	0.6870

Correlation of BMI SDS with respective endocrinological parameters

**Figure 1:**
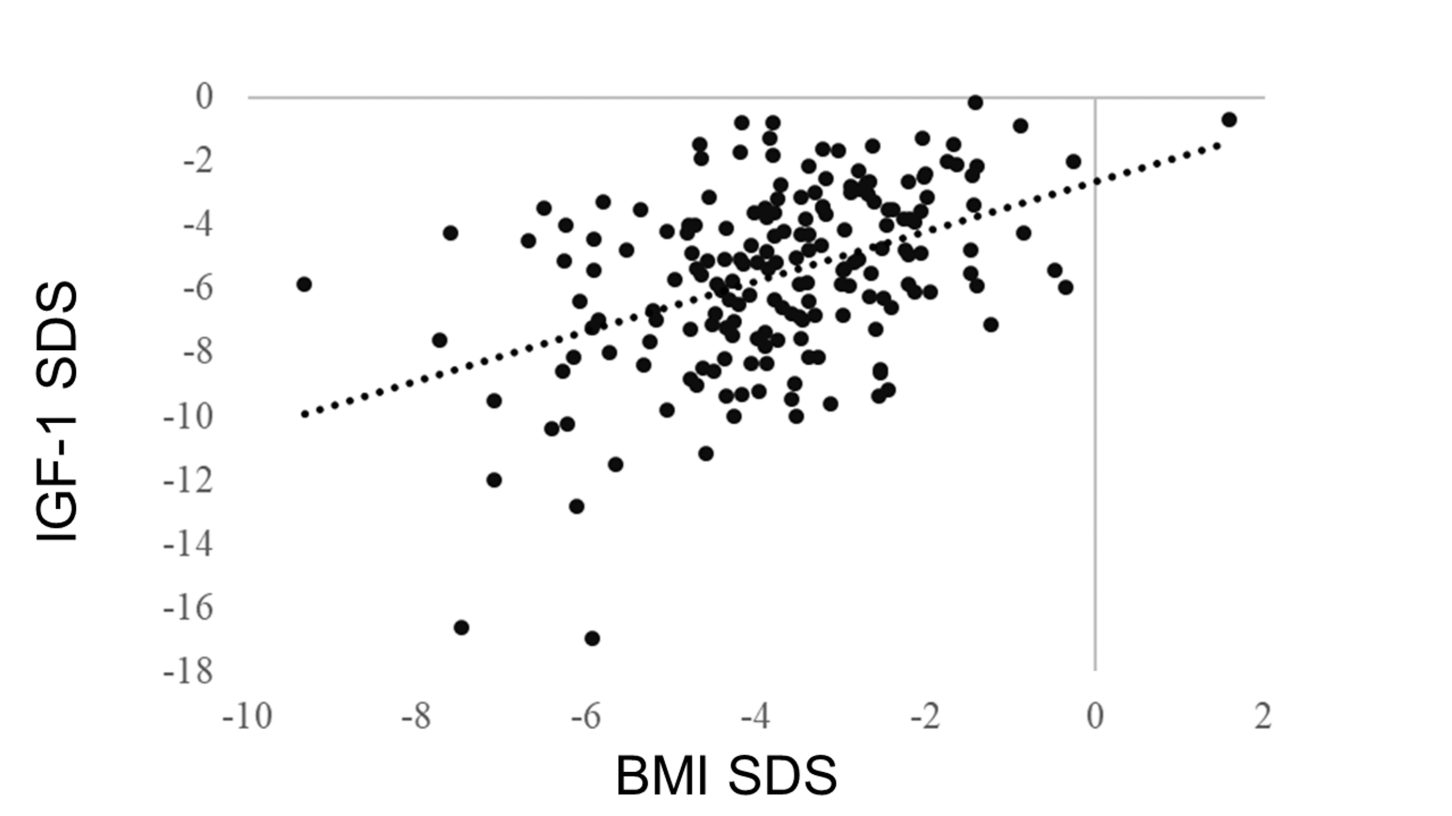
Correlation between IGF-1 SDS and BMI SDS. IGF-1 SDS was positively correlated with BMI SDS (R = 0.405, p < 0.0001). IGF-1: insulin-like growth factor I; SDS: standard deviation score; BMI: body mass index

**Figure 2:**
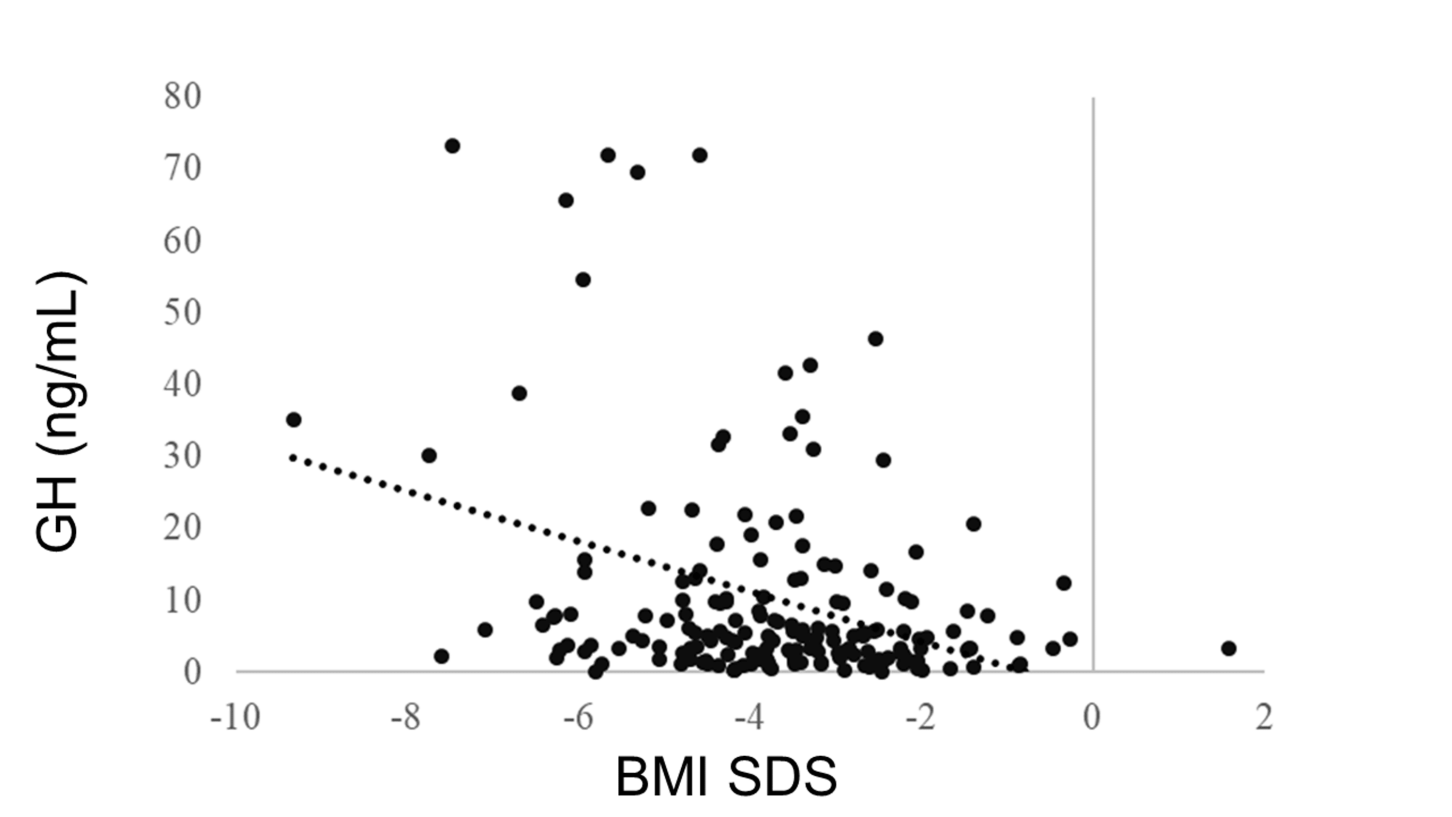
Correlation between GH and BMI SDS. GH was negatively correlated with BMI SDS (R = –0.241, P = 0.0096). GH: growth hormone; BMI: body mass index; SDS: standard deviation score

**Figure 3:**
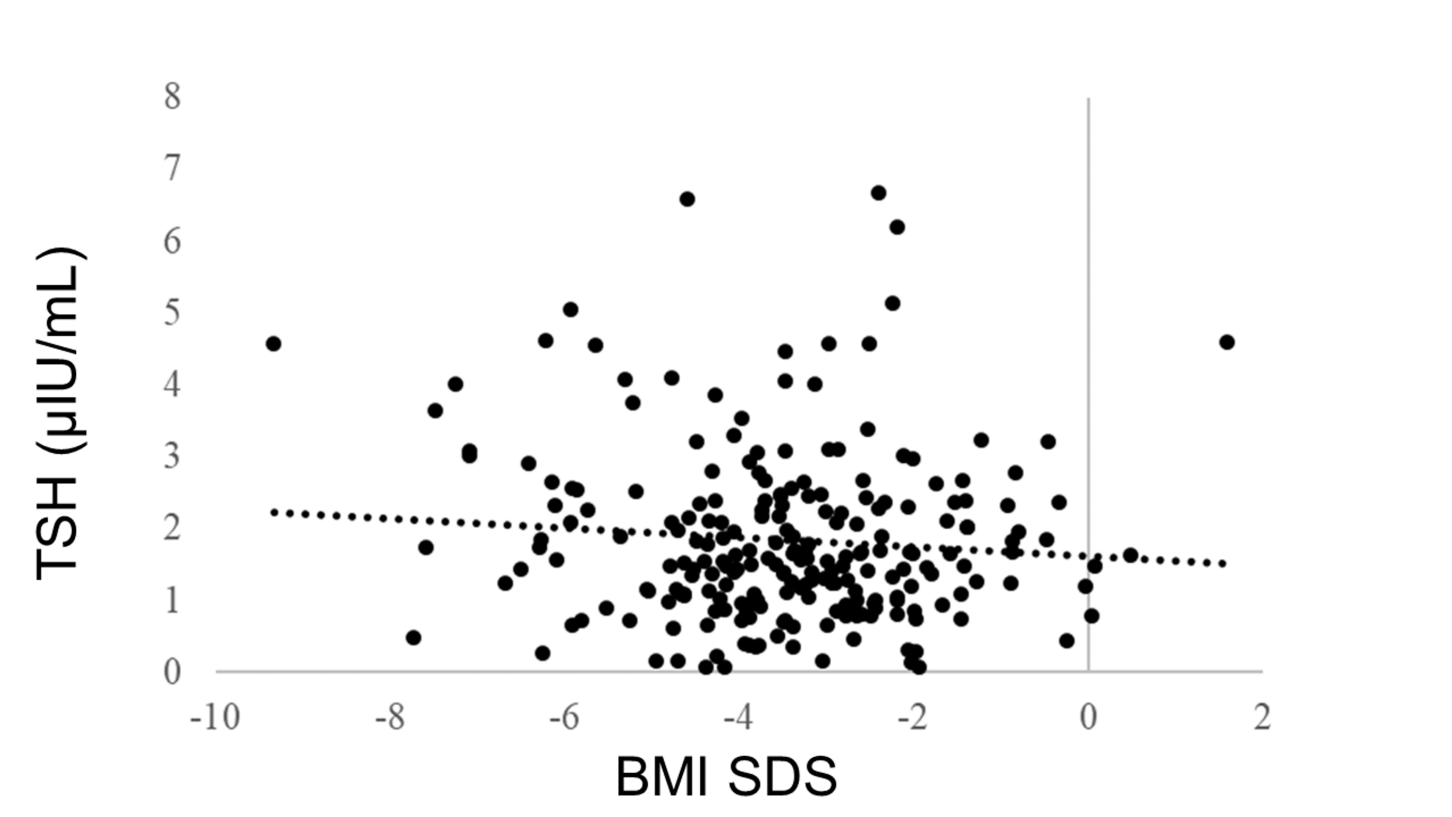
Correlation between TSH and BMI SDS. No correlation was found between BMI SDS and TSH (R = −0.043, p = 0.5180). TSH: thyroid-stimulating hormone; BMI: body mass index; SDS: standard deviation score

**Figure 4:**
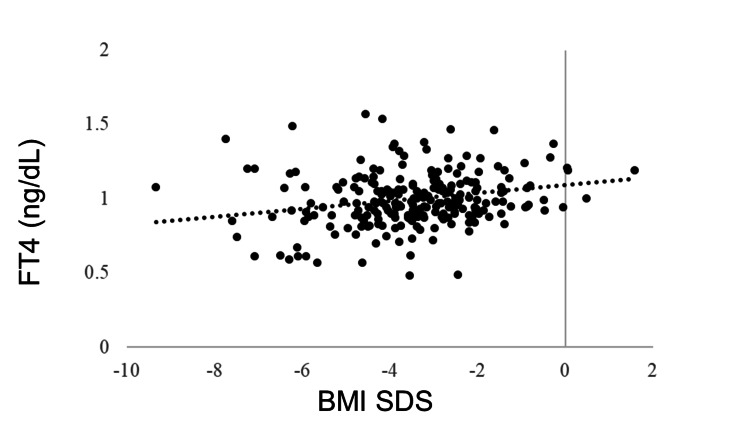
Correlation between FT4 and BMI SDS. BMI SDS and FT4 were positively correlated (R = 0.205, p = 0.0018). BMI: body mass index; SDS: standard deviation score; FT4: free thyroxine

**Figure 5:**
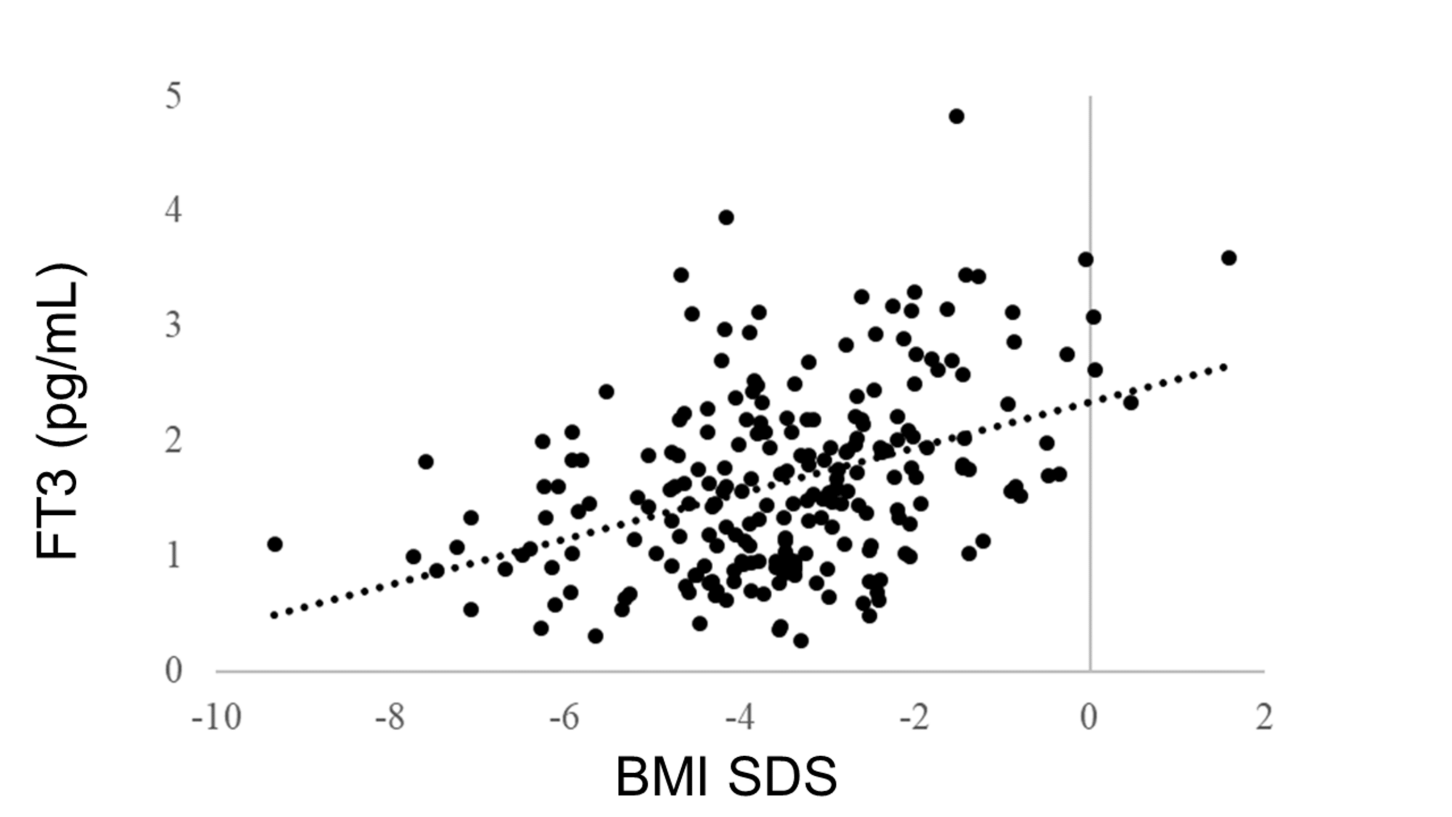
Correlation between FT3 and BMI SDS. FT3 was positively correlated with BMI SDS (R = 0.381, p < 0.0001). FT3: free triiodothyronine; BMI: body mass index; SDS: standard deviation score

**Figure 6:**
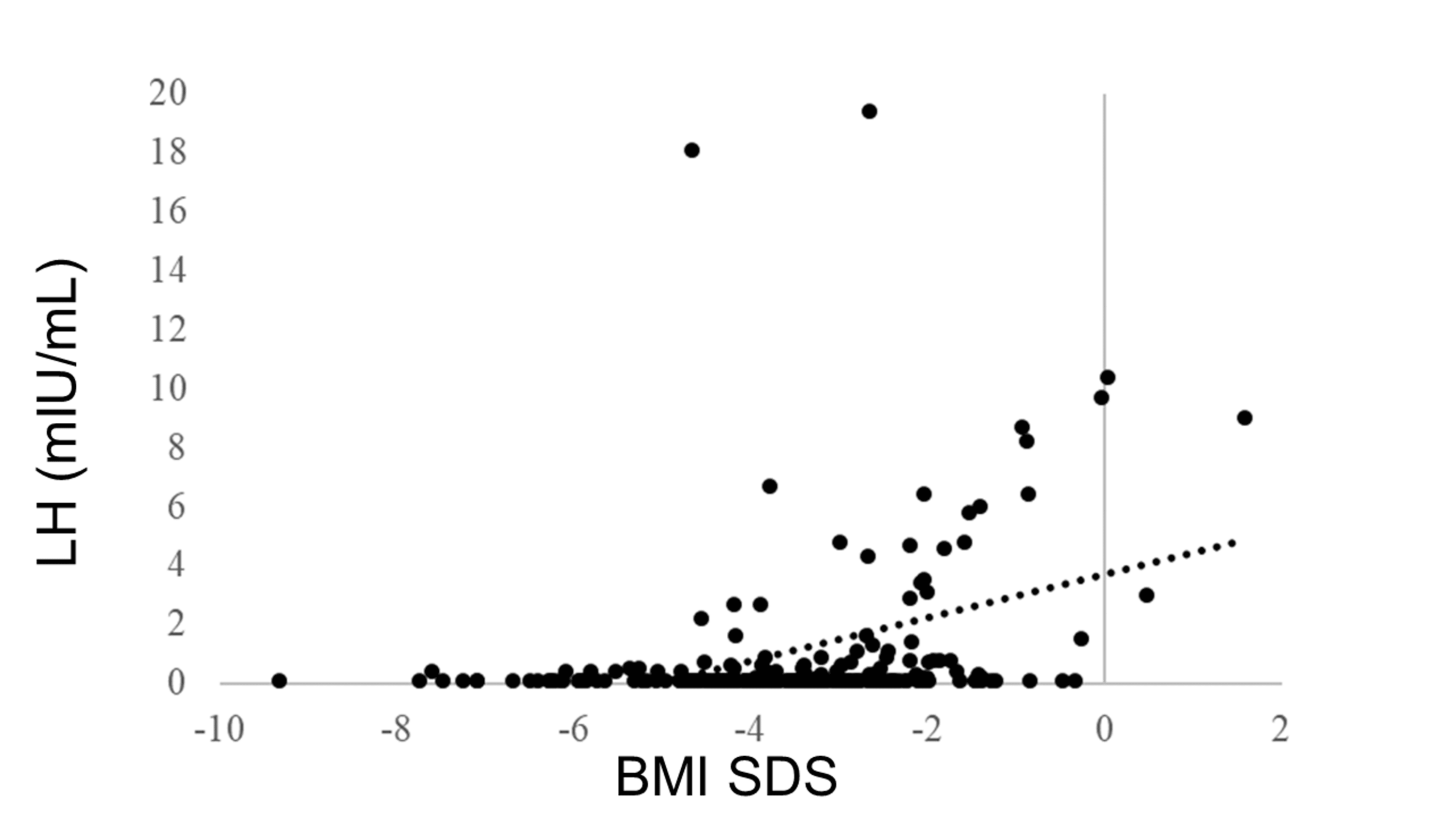
Correlation between LH and BMI SDS. LH levels were positively correlated with BMI SDS (R = 0.365, p < 0.0001). LH: luteinizing hormone; BMI: body mass index; SDS: standard deviation score

**Figure 7:**
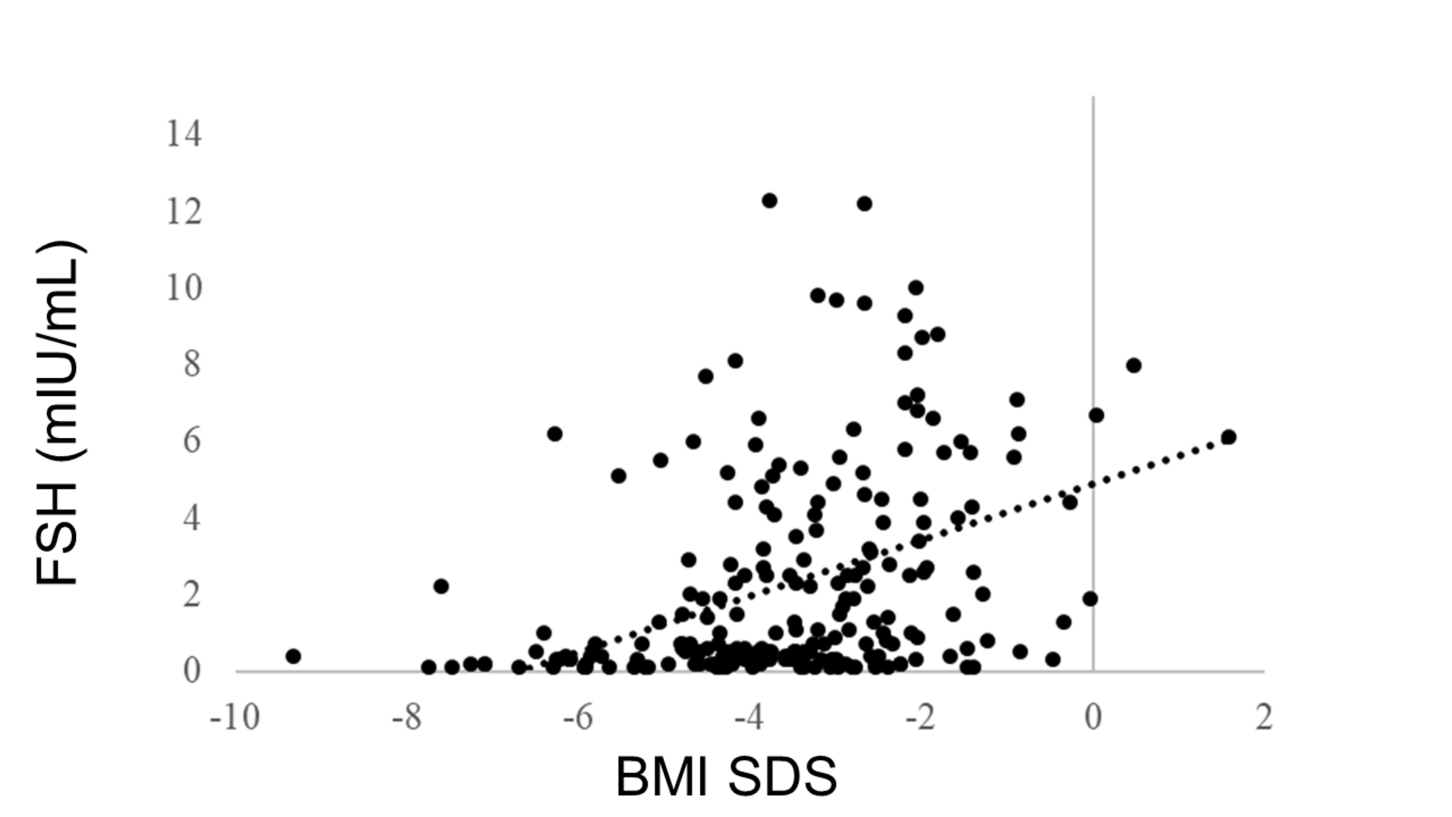
Correlation between FSH and BMI SDS. FSH levels were positively correlated with BMI SDS (R = 0.409, p < 0.0001). BMI: body mass index; SDS: standard deviation score; FSH: follicle-stimulating hormone

**Figure 8:**
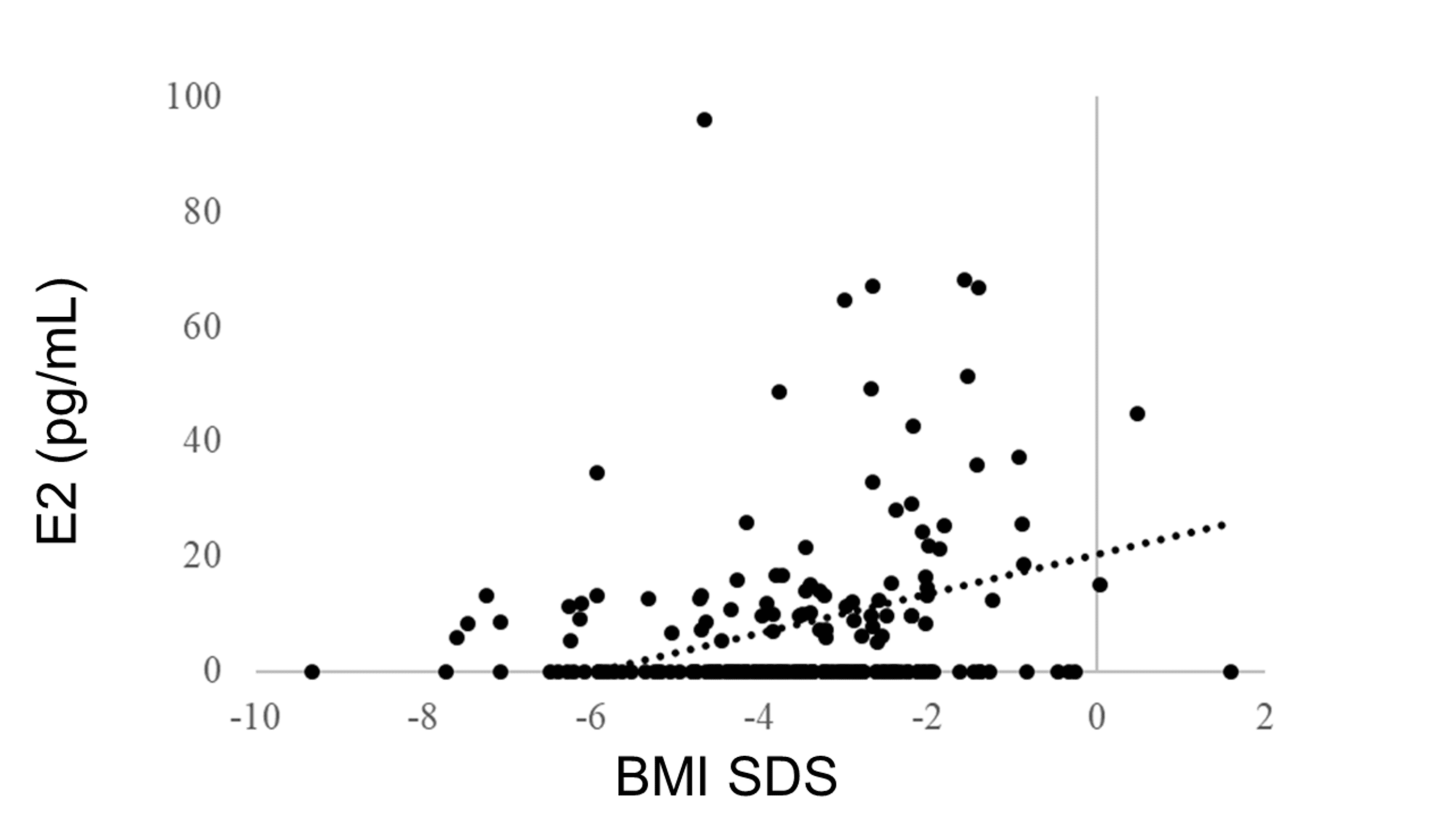
Correlation between E2 and BMI SDS. BMI SDS was positively correlated with E2 (R = 0.227, p = 0.0006). BMI: body mass index; SDS: standard deviation score; E2: estradiol

**Figure 9:**
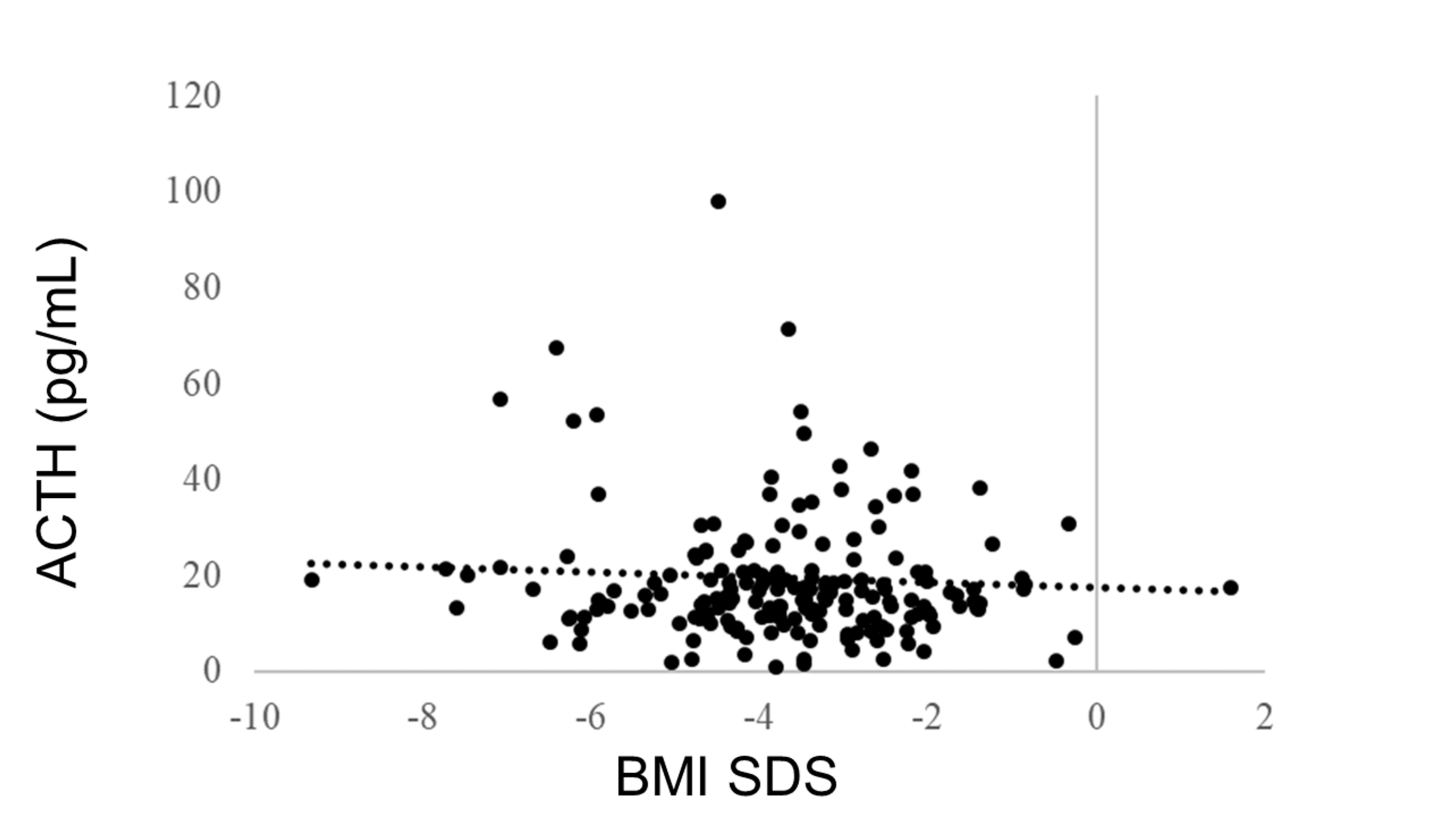
Correlation between ACTH and BMI SDS. ACTH was not correlated with BMI SDS (R = −0.031, p = 0.6770). BMI: body mass index; SDS: standard deviation score; ACTH: adrenocorticotropic hormone

**Figure 10:**
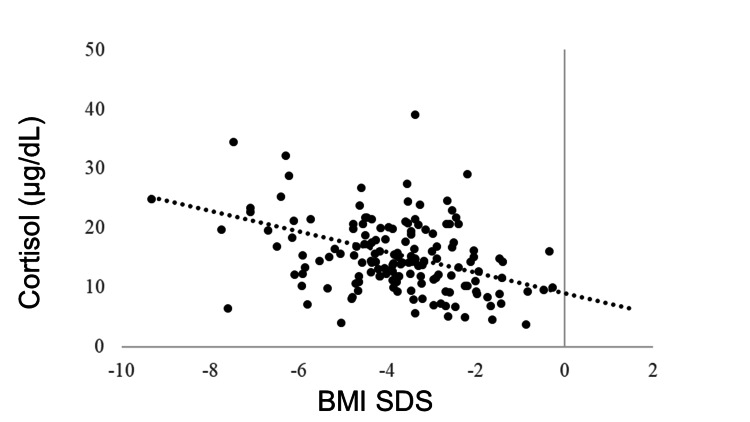
Correlation between cortisol and BMI SDS. Cortisol was negatively correlated with BMI SDS (R = −0.321, p <0.0001). BMI: body mass index; SDS: standard deviation score

**Figure 11:**
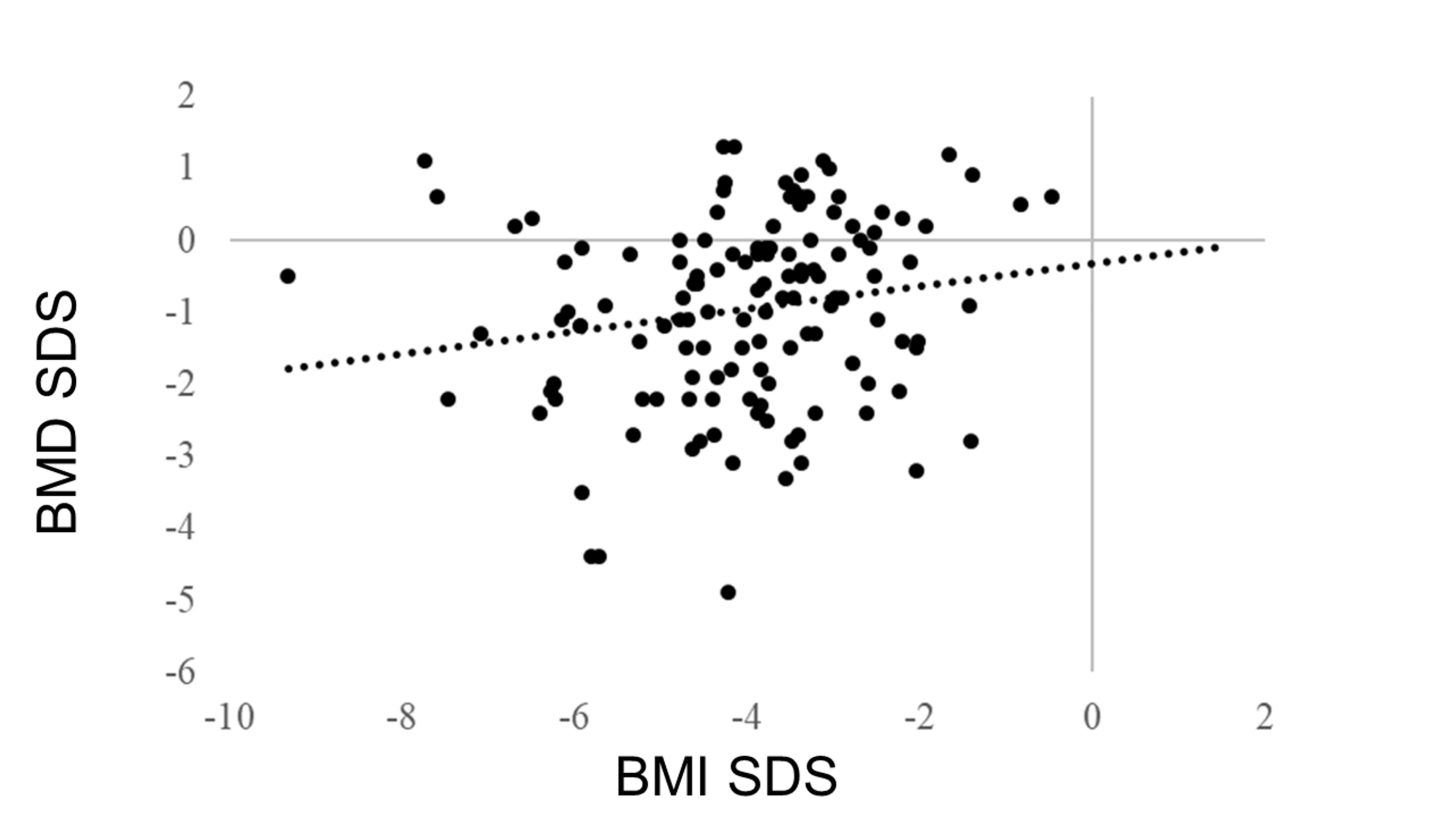
Correlation between BMD SDS and BMI SDS. BMD SDS was positively correlated with BMI SDS (R = 0.222, p = 0.0107). BMD: bone mineral density; SDS: standard deviation score; BMI: body mass index

IGF-1 SDS was positively correlated with BMI SDS (R = 0.405, p < 0.0001). GH was negatively correlated with BMI SDS (R = −0.278, p < 0.0001). No correlation was observed between BMI SDS and TSH (R = −0.043, p = 0.5180), but BMI SDS and FT4 were positively correlated (R = 0.205, p = 0.0018). FT3 was positively correlated with BMI SDS (R = 0.381, p < 0.0001). The LH and FSH levels were positively correlated with BMI SDS (R = 0.365, p < 0.0001 and R = 0.409, p < 0.0001, respectively). BMI SDS was positively correlated with E2 (R = 0.227, p = 0.0006). ACTH was not correlated with BMI SDS (R = −0.031, p = 0.6770), whereas cortisol was negatively correlated with BMI SDS (R = −0.321, p < 0.0001) (Table 3, Figures 1-11).

**Table 3:** Correlation with body mass index standard deviation score. ACTH: adrenocorticotropic hormone; BMD: bone mineral density; BMI: body mass index; E2: estradiol; FSH: follicle-stimulating hormone; FT3: free triiodothyronine; FT4: free thyroxine; GH: growth hormone; IGF-1: insulin-like growth factor I; LH: luteinizing hormone; SDS: standard deviation score; TSH: thyroid-stimulating hormone p-values of <0.05 were considered statistically significant. p-values of <0.0001 are highly statistically significant. *p < 0.05; **p < 0.0001

	Correlation coefficient (R) with BMI SDS	p-value
IGF-1 SDS	0.405	<0.0001**
GH	−0.241	0.0096*
TSH	−0.043	0.5180
FT4	0.205	0.0018*
FT3	0.381	<0.0001**
LH	0.365	<0.0001**
FSH	0.409	<0.0001**
E2	0.227	0.0006*
ACTH	−0.031	0.6770
Cortisol	−0.321	<0.0001**
BMD	0.222	0.0107*

Correlation between GH and IGF-1 SDS

**Figure 12:**
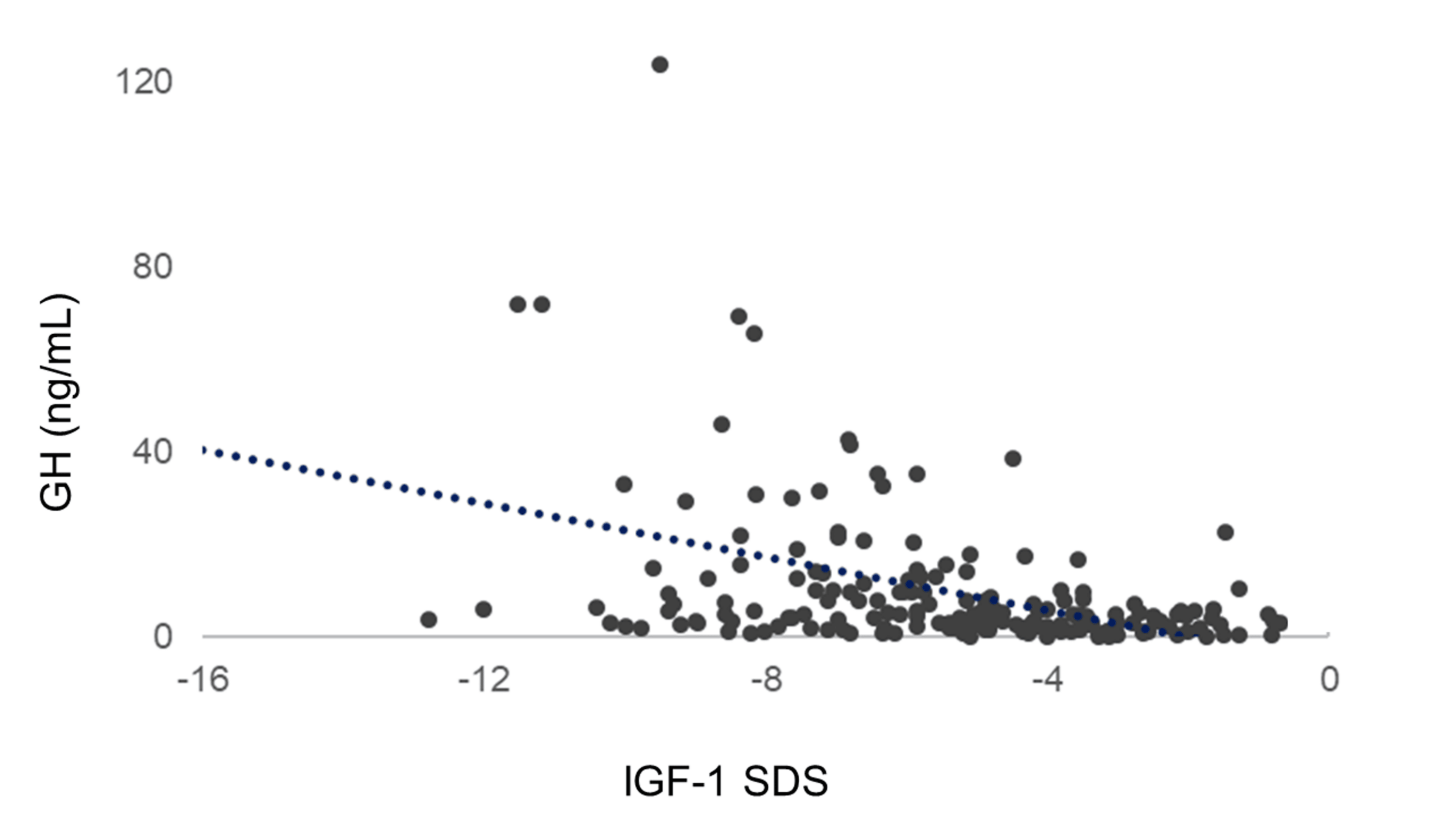
Correlation between GH and IGF-1 SDS A negative correlation was found between IGF-1 SDS and GH (R = −0.424, p < 0.0001). GH: growth hormone; IGF-1: insulin-like growth factor I; SDS: standard deviation score

A negative correlation was found between IGF-1 SDS and GH (R = −0.424, p < 0.0001). Despite high GH secretion, IGF-1 levels were low, and GH resistance was observed (Figure 12).

## Discussion

This report describes the neurogenic and endocrinological features in children with AN. We observed the presence of GH resistance and hypothalamic amenorrhea, similar to a previous report in adults. However, ACTH elevation was not observed, which contrasts with findings in adult cases.

Patients with AN that develop during the growth spurt period may show a slowing of growth and weight loss that deviates from the standard growth curve, resulting in a final height less than the estimated normal value for their age group [12]. Patients with AN recover their height growth and their estimated final height upon weight restoration; however, the final height is lower when weight recovery is delayed. Patients may take several years to recover, and the process is often gradual. Catch-up growth is highly variable, ranging from complete recovery to failure to gain any height [13]. Under low nutritional conditions, such as starvation, GH secretion increases in the short term but decreases in the long term. In the present study, despite the high GH levels in AN, IGF-1 levels were reduced, which is thought to indicate a GH-resistant state in the liver [4-6]. Therefore, our study revealed that, similar to adults with AN, children with AN showed GH resistance.

Women with AN have higher GH burst frequency, mass, and duration than healthy controls, resulting in a fourfold increase in daily pulsatile GH secretion and a 20-fold increase in basal GH secretion [14]. A potential mechanism for GH resistance may involve elevated levels of fibroblast growth factor 21, which inhibits the intracellular effects of GH, including the production of IGF-1 by the liver [15]. GH resistance may be mediated, in part, by low insulin levels during chronic undernutrition, which downregulates hepatic GH receptor expression [16]. However, contradictory results regarding the efficacy of GH treatment for AN indicate the complex nature of GH mechanisms [17].

In our report, dysregulation of the hypothalamic-pituitary-gonadal (HPG) axis was observed in childhood-onset AN. Notably, amenorrhea was present in at least 90% of the patients, even in those without weight loss or mild disease. The mechanisms underlying the disruption of the HPG axis in chronic undernutrition include low leptin levels and hypercortisolemia due to chronic stress [18].

Individuals with AN have significantly lower bone mineral density (BMD) compared to normal-weight individuals [19]. BMD increases with the onset of secondary sexual characteristics, peaking at approximately 16 years of age. In general, peak bone mass (PBM) is maintained until the late 20s and then gradually declines. PBM acquisition is primarily related to gonadal hormones, and supporting factors include IGF-1, weight gain, and weight-bearing exercises [20]. Therefore, hypogonadotropic hypogonadism, low IGF-1 levels, and weight loss may contribute to low PBM in patients with AN. In such patients, the levels of osteocalcin and bone-type alkaline phosphatase, which are markers of bone formation, decreased. However, urinary N-terminal telopeptide, a marker of bone resorption, increased [21]. Overall, these changes indicate abnormalities in bone metabolism. Unexpectedly, in our study, we found only a weak correlation between the BMD SDS and BMI SDS scores (R = 0.222, p = 0.0107). There are several possible explanations for this lack of correlation. First, all data were collected during the initial examination. Second, the time from disease onset was short in a large proportion of cases. Third, the study did not involve a longitudinal analysis. During the long-term course of the disease, some patients with refractory disease exhibited a marked decrease in the BMD SDS scores (data not shown); therefore, long-term follow-up is essential to reveal the relationship between BMD and BMI.

Physiological stress, including chronic undernutrition, activates the HPA axis. In at least one-third of women with AN, the HPA axis is chronically stimulated [19]. This dysfunction in the HPA axis may be attributed to the stress from chronic nutritional deprivation and may be a means of maintaining euglycemia in patients with AN [22]. Elevated 24-hour urinary free cortisol levels [23], overnight mean serum cortisol levels [19], cortisol response to synuclein administration [24], dexamethasone suppression [25], and midnight salivary cortisol levels [26] are commonly, although not universally, elevated in women with AN. In our study, cortisol levels increased with increasing disease severity, although they remained within the normal range. In contrast, the ACTH levels remained almost constant regardless of disease severity. Unlike previous studies, this study did not evaluate the time of blood collection, diurnal variations, or urinary cortisol levels during urine storage. Therefore, although cortisol was positively correlated with BMI SDS, we cannot conclusively determine that hypercortisolemia was uncommon in children with AN.

The hypothalamic-pituitary-thyroid axis is a key regulator of metabolic processes [27]. Hypothyroidism symptoms, such as bradycardia, hypothermia, hypotension, dry skin, and slow metabolic rate, are prevalent in patients with AN. Decreased conversion of T4 to T3 reduces resting energy expenditure [28], while increased conversion of T4 to reverse T3, which is the metabolically inactive form of T3, increases energy expenditure. In a more severe state, TSH levels in the anterior pituitary and free T4 levels in the thyroid may fall to the low-normal range due to the general suppression of the hypothalamic-pituitary-thyroid axis. This thyroid function pattern is referred to as a non-thyroidal illness or low T3 syndrome [29]. In this study, we found that the TSH levels remained constant regardless of severity, T3 had a positive correlation with BMI SDS, and T4 had a weak positive correlation with BMI SDS. Weight recovery results in an increase in the total T3 levels, while no significant change was observed in the serum TSH or T4 levels [30]. Therefore, monitoring T3 levels during the treatment course may be useful for assessing metabolic adaptation and recovery of the target weight.

The current study had some limitations. First, this was a retrospective study, and the timing of blood tests and meals varied. Additionally, as only a single measurement of hormone levels was obtained at the initial evaluation, longitudinal changes with BMI recovery could not be assessed. Second, as this was a single-center study, there was an inherent bias for region, age, and severity. Third, bias was present because blood tests were not performed in some mild cases. Fourth, data on the relationships between final height, menstruation, and BMD were absent. Despite these limitations, to the best of our knowledge, this is the first large-scale study on the endocrine characteristics of childhood-onset AN in Japan.

## Conclusions

In conclusion, we investigated the characteristics of 246 patients with childhood-onset eating disorders. The patients were predominantly female, and 97.5% of them were amenorrheic. Although IGF-1 SDS decreased with increasing severity, GH value increased with increasing severity, indicating GH resistance, consistent with adult cases. FT4 and FT3 levels decreased with increasing severity. LH, FSH, and E2 decreased with increasing severity, and central hypogonadism was observed, consistent with adult cases. In the adrenal system, the ACTH level was almost constant regardless of severity, while the cortisol level increased with severity progression, which is inconsistent with adult cases.
